# Target Fitting Method for Spherical Point Clouds Based on Projection Filtering and K-Means Clustered Voxelization

**DOI:** 10.3390/s24175762

**Published:** 2024-09-04

**Authors:** Zhe Wang, Jiacheng Hu, Yushu Shi, Jinhui Cai, Lei Pi

**Affiliations:** 1 Key Laboratory of In-Situ Metrology, Ministry of Education, China Jiliang University, Hangzhou 310018, China; 18968114109@163.com (Z.W.); caijinhui@cjlu.edu.cn (J.C.); 2 National Institute of Metrology, Beijing 102200, China; shiys@nim.ac.cn (Y.S.); pilei@nim.ac.cn (L.P.)

**Keywords:** sphere target fitting (STF), three-dimensional point data projection, K-Means clustering, CT measurement

## Abstract

Industrial computed tomography (CT) is widely used in the measurement field owing to its advantages such as non-contact and high precision. To obtain accurate size parameters, fitting parameters can be obtained rapidly by processing volume data in the form of point clouds. However, due to factors such as artifacts in the CT reconstruction process, many abnormal interference points exist in the point clouds obtained after segmentation. The classic least squares algorithm is easily affected by these points, resulting in significant deviation of the solution of linear equations from the normal value and poor robustness, while the random sample consensus (RANSAC) approach has insufficient fitting accuracy within a limited timeframe and the number of iterations. To address these shortcomings, we propose a spherical point cloud fitting algorithm based on projection filtering and K-Means clustering (PK-RANSAC), which strategically integrates and enhances these two methods to achieve excellent accuracy and robustness. The proposed method first uses RANSAC for rough parameter estimation, then corrects the deviation of the spherical center coordinates through two-dimensional projection, and finally obtains the spherical center point set by sampling and performing K-Means clustering. The largest cluster is weighted to obtain accurate fitting parameters. We conducted a comparative experiment using a three-dimensional ball-plate standard. The sphere center fitting deviation of PK-RANSAC was 1.91 μm, which is significantly better than RANSAC’s value of 25.41 μm. The experimental results demonstrate that PK-RANSAC has higher accuracy and stronger robustness for fitting geometric parameters.

## 1. Introduction

Computed tomography (CT), derived from X-ray imaging technology, emerged in the 1970s. CT is an imaging technology that uses high-energy rays to penetrate objects to obtain projections and reconstruct multi-angle projections, using various algorithms to obtain object tomographic information [1]. CT has the advantages of high spatial resolution and easy visualization and plays a significant role in the medical field. From the late 1970s to the 1990s, with the rapid development of digital image processing technology and microcomputer systems, CT technology has expanded from the medical field to industrial fields such as non-destructive testing, reverse engineering, and material organization analysis, has been widely used in agriculture, forestry, and geophysics [2], and remains a popular research topic.

Especially in the field of industrial measurement, CT has developed rapidly, owing to its non-contact and penetrable measurement characteristics, avoiding contact deformation in three-dimensional coordinate measuring machines [3] and enabling the measurement of the internal information of objects [4]. Spherical objects represent an important type of object in CT measurement as they are often used to construct ball-plate standards to trace and calibrate industrial CT instruments, as well as in point cloud registration and other tasks. The spherical radius and spherical center coordinates are key measurement parameters, and the measurement process of CT for spherical objects is relatively intuitive. A spherical object can be placed at the rotation center of the CT turntable to obtain projection data. After processing with a three-dimensional reconstruction algorithm and gridding algorithm, the point cloud data of the spherical object can be obtained. Then, point cloud data processing can be performed to obtain measurement parameters such as spherical coordinates and radius. Researchers have proposed a variety of fitting methods for point cloud targets, which can largely be divided into two categories, namely, the least squares (LS) and numerical optimization (NO) methods [5].

LS is a mathematical optimization modeling method that searches for the best function match for given data by minimizing the sum of squared errors to achieve optimal parameter estimation. In 2005, Reshetyuk [6] used the definition formula of a sphere in three-dimensional space combined with the LS method to construct a linear equation system and obtained fitting parameters by solving this equation system. Subsequently, Lu [7] applied the total LS (TLS) algorithm to spherical point cloud fitting. In addition to minimizing the sum of the perpendicular distances from the point to the surface of the sphere, the TLS algorithm also attempts to minimize the sum of the squared residuals of each point. Le [8] used the truncated LS method to solve the robustness problem of data fitting. The truncated LS method introduces a truncation function to handle outliers, thereby improving the robustness of fitting. In 2015, Bektas [9] used the orthogonal distance LS method to fit an ellipsoid, accounting for the actual distance from the sample point to the fitting surface, rather than simply summing the squared errors of all points. Subsequently, Liu [10] proposed an algorithm combining nonlinear LS (NLS) and AD-random sample consensus (RANSAC) to detect spherical targets and achieve rapid fitting of such targets in mobile terrestrial laser scanning point cloud data. Wu [11] combined RANSAC with the LS method and proposed PC-RANSAC, which first calculates the principal curvature of point cloud data to implement a principal curvature constraint on random sampling points, and then uses the LS method to estimate geometric parameters. Tao [12] proposed a constrained weighted LS algorithm to search for the best plane to achieve iterative close point registration, diversifying the application of fitting methods in the field of point cloud registration. Fei [13] proposed a five-parameter constrained LS method for small-angle spherical surface fragments. In this method, even if the segment angle approaches 1°, a certain degree of accuracy is guaranteed. In recent research, Li [14] proposed an adaptive cylindrical fitting algorithm based on Robust Principal Component Analysis (RPCA). This algorithm was experimentally validated for its effectiveness and robustness across various datasets, demonstrating superior performance in handling noisy data. Zong [15] introduced a three-dimensional line fitting algorithm based on recursive weighted LS, aimed at significantly reducing the impact of boundary noise on the fitting results. Meanwhile, Zheng [16] developed a point cloud data processing method based on iterative residual fitting. This method first performs LS fitting on all points to calculate the residual values after the initial fitting, and then selects the points with residuals below a specified threshold to form a new point cloud. Subsequently, the remaining point cloud undergoes a second LS fitting, and the residual values from this second fitting are calculated. This process is repeated until the conditions for terminating the iteration are met, thereby achieving precise processing of the point cloud data. In short, the goal of the LS method and its variants is to minimize the error between model prediction values and observation values to obtain a more accurate geometric parameter solution.

The NO approach aims to maximize or minimize a specific evaluation criterion by identifying optimal model parameters [17]. Many estimation algorithms can be used to solve the spherical target fitting (STF) problem. Based on whether there are feature constraints in the estimation algorithm, NO approaches can be divided into two categories: ordinary NO and feature-constrained NO [5]. Three-dimensional point cloud models typically contain a large amount of point information, and performing three-dimensional Hough transformation consumes significant computing resources and time. Additionally, owing to the sensitivity of the Hough transform to noise and poor robustness [18,19], it is not widely used in three-dimensional point cloud fitting. In 2007, Schnabel [20] used RANSAC to detect the shapes of unorganized point clouds. Specifically, RANSAC was used to derive a geometric model by sampling within a set number of iterations and using a predefined threshold to judge whether points meet certain requirements to extract an optimal model [21,22]. Subsequently, Schnabel [23,24] used the MELSAC and MSAC algorithms to detect point clouds of various shapes. MSAC is a model based on M-estimation that aims to obtain the best fit in each iteration, whereas MLESAC is based on maximum likelihood estimation. Kang [25] used threshold-independent Bayesian sampling (BAYSAC) to implement plane detection. Specifically, they used point cloud downsampling to retain feature boundaries and BAYSAC to fit planes [26,27]. Shi [28] proposed a limited random search algorithm based on point cloud data and the geometric features of spherical targets. Using parameter estimation, this method realizes SFT from a probabilistic statistical perspective. Additionally, Shi [29] proposed an adaptive grid search algorithm that fully leverages the geometric features of spherical targets and obtains the best fitting parameters through a limited number of iterative optimizations. Bin [30] proposed a nonlinear Gauss–Helmert (NGH) model to describe the mathematical model of point cloud formation and then proposed a novel point cloud fitting method based on the robust NGH model. Unlike previous approaches, this fitting method accounts for all random errors in various linear and nonlinear fitting problems, which can effectively reduce the influence of interference points. Shu [31] proposed an improved RANSAC algorithm that integrates Newton’s iterative method for precise cylindrical fitting of rebar and corrugated pipes. This method enhances the convergence speed and fitting accuracy by introducing adaptive thresholds and constraints on the minimum number of inliers. Zhang [32] developed a curvature consistency sphere detection (CCSD) algorithm for sphere recognition in light detection and ranging (LiDAR) point clouds. The CCSD employs RANSAC for sphere fitting during the final detection stage. It also introduces a mixed voting mechanism to calculate the observation error of candidate spheres based on curvature consistency, inlier support rate, and size deviation. This improves the accuracy of sphere fitting. Singh [33] utilized an enhanced progressive sample consensus (PROSAC) algorithm for plane fitting of point cloud data to assess terrain complexity. PROSAC is an improved version of RANSAC that enhances the efficiency of the algorithm by incorporating prior knowledge to guide the sampling process. Furthermore, Li [34] proposed a method for extracting pavement cracks based on three-dimensional laser point clouds. This method combines the M-estimator sample consensus (MSAC) algorithm with the K-nearest neighbor (KNN) algorithm. Initially, the MSAC algorithm fits the preprocessed point cloud to a plane. Then, based on the characteristic that crack points are primarily located below the pavement, it separates the crack point cloud from the pavement texture points. In summary, the main goal of the NO approach is to vary the sampling method or searching mode within a limited number of iterations to obtain an optimal solution.

Additionally, Simon Burkhard [35] proposed a method for fitting the projected centers of spheres in cone beam X-ray imaging. To model the edge of the sphere with sub-pixel resolution, the tangent circle of the sphere was decomposed three times. Compared with elliptical target fitting, this method reduces the number of unknown variables and has advantages for numerical calculation. Hong [36] combined the random forest model with machine learning, and this method demonstrated excellent performance in terms of fitting accuracy and generalization capability.

In summary, LS and its variants and NO algorithms are all effective ways to fit point clouds. However, these algorithms still have shortcomings in terms of further improving accuracy and robustness. LS obtains fitting parameters by solving a system of linear equations. When abnormal points are present, the solution obtained will deviate significantly from the actual value. Additionally, when an equation takes the x and y coordinates of point cloud data as independent variables, it only considers the error of the observation vector (i.e., vector z), implying that it ignores the error in the x and y coordinates in the coefficient matrix. The main shortcoming of the NO approach is that some algorithms are sampled within a certain number of iterations. If a more accurate model is to be obtained, an exact threshold and a larger number of iterations are required. RANSAC is a robust and commonly used algorithm; however, owing to the randomness of sampling, it is difficult to guarantee the accuracy of results.

To overcome the limitations described above, this paper proposes a spherical point cloud fitting algorithm (PK-RANSAC) based on projection filtering and K-means clustering. We selected several existing algorithms for comparison with PK-RANSAC. Experimental results demonstrate that the proposed algorithm combines the advantages of LS and NO algorithms, can improve both robustness and accuracy, and yields better fitting parameters.

## 2. Materials and Methods

**Figure 1:**
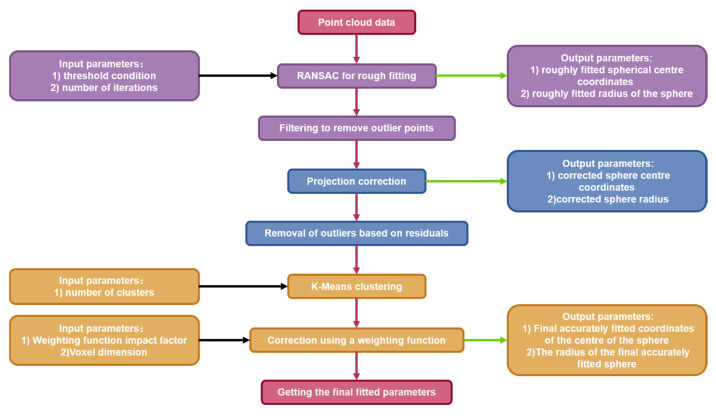
Overall process of the proposed algorithm (first step (the purple part): RANSAC rough fitting; second step (the blue part): projection filter correction; third step (the yellow part): accurate fitting to obtain parameters).

This section introduces the implementation process of PK-RANSAC, which is mainly divided into three steps: RANSAC rough fitting, projection filter correction, and precise determination of fitting parameters. In the RANSAC rough fitting process, preliminary fitting of the sphere parameters is performed. In the projection correction process, the correction value is obtained by considering two projections in opposite directions. In the process of accurately obtaining fitting parameters, fitting results are obtained through K-means clustering and a weighting function. The algorithm solution flow is illustrated in Figure 1.

### 2.1. RANSAC Rough Estimation

RANSAC is widely used for fitting geometric objects such as planes, spheres, and cylinders. Its speed and robustness represent significant advantages over the LS method. In three-dimensional space, four non-coplanar points define a sphere. The formula for defining a sphere with four points in three-dimensional space is presented in Equation (1). Random sampling is performed on point cloud data, and the four non-coplanar points selected are denoted as $A(A_{X},A_{Y},A_{Z})$, $B(B_{X},B_{Y},B_{Z})$, $C(C_{X},C_{Y},C_{Z})$, and $D(D_{x},D_{y},D_{z})$. Substituting these four points into Equation (1) yields a system of linear equations.

(1) $${\left(X_{Sphere}-X\right)}^{2}+{\left(Y_{Sphere}-Y\right)}^{2}+{\left(Z_{Sphere}-Z\right)}^{2}=R_{Sphere}^{2}$$

After solving these equations, the following related parameters *D* (Equation (2)), *P* (Equation (3)), *Q* (Equation (3)), and *R* (Equation (3)) can be obtained. Then, *DX* (Equation (4)), *DY* (Equation (5)), and *DZ* (Equation (6)) can be obtained based on these parameters.

(2) $$D=\left(\begin{array}{ccc} \left(A_{x}-B_{x}\right) & \left(A_{y}-B_{y}\right) & \left(A_{z}-B_{z}\right)\\ \left(C_{x}-D_{x}\right) & \left(C_{y}-D_{y}\right) & \left(C_{z}-D_{z}\right)\\ \left(B_{x}-C_{x}\right) & \left(B_{y}-C_{y}\right) & \left(B_{z}-C_{z}\right) \end{array}\right)$$

(3) $$\left\{\begin{array}{l} P=\frac{1}{2}(A_{x}^{2}-B_{x}^{2}+A_{y}^{2}-B_{y}^{2}+A_{z}^{2}-B_{z}^{2})\\ Q=\frac{1}{2}(C_{x}^{2}-D_{x}^{2}+C_{y}^{2}-D_{y}^{2}+C_{z}^{2}-D_{z}^{2})\\ R=\frac{1}{2}(B_{x}^{2}-C_{x}^{2}+B_{y}^{2}-C_{y}^{2}+B_{z}^{2}-C_{z}^{2}) \end{array}\right.$$

(4) $$DX=\left(\begin{array}{ccc} P & \left(A_{y}-B_{y}\right) & \left(A_{z}-B_{z}\right)\\ Q & \left(C_{y}-D_{y}\right) & \left(C_{z}-D_{z}\right)\\ R & \left(B_{y}-C_{y}\right) & \left(B_{z}-C_{z}\right) \end{array}\right)$$

(5) $$DY=\left(\begin{array}{ccc} \left(A_{x}-B_{x}\right) & P & \left(A_{z}-B_{z}\right)\\ \left(C_{x}-D_{x}\right) & Q & \left(C_{z}-D_{z}\right)\\ \left(B_{x}-C_{x}\right) & R & \left(B_{z}-C_{z}\right) \end{array}\right)$$

(6) $$DZ=\left(\begin{array}{ccc} \left(A_{x}-B_{x}\right) & \left(A_{y}-B_{y}\right) & P\\ \left(C_{x}-D_{x}\right) & \left(C_{y}-D_{y}\right) & Q\\ \left(B_{x}-C_{x}\right) & \left(B_{y}-C_{y}\right) & R \end{array}\right)$$

According to Cramer’s rule, the sphere radius *R* and the three coordinate parameters $X_{Sphere}$, $Y_{Sphere}$, and $Z_{Sphere}$ are obtained as shown in Equation (7).

(7) $$\left\{\begin{array}{l} X_{Sphere}=DX/D\\ Y_{Sphere}=DY/D\\ Z_{Sphere}=DZ/D\\ R=dis(Centre_{Sphere},A) \end{array}\right.$$

The main concept of RANSAC is to determine an appropriate threshold within a limited number of iterations and use four non-coplanar points to obtain the parameters of the sphere model as described earlier. Then, it is determined whether each point meets the threshold requirements. If so, a point is called an inlier; otherwise, it is called an outlier. The sphere model with the most inliers is identified as the optimal model within the maximum number of iterations, and a filtering algorithm is used to remove abnormal points. The main goal of the first step is to obtain preliminary fitting parameters.

### 2.2. Projection Correction

**Figure 2:**
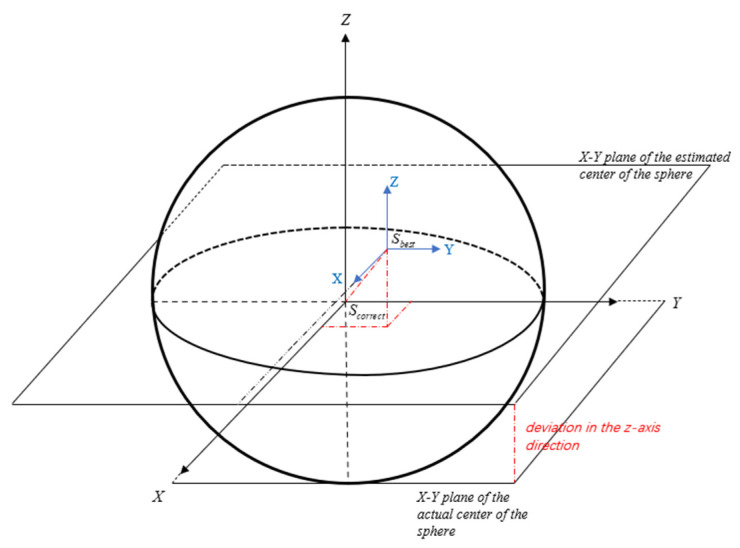
Schematic of the actual $S_{correct}$ and roughly estimated center of the sphere $S_{best}$ (the blue coordinate system is the rough estimate of the center of the sphere, while the black coordinate system is the actual center of the sphere).

The center of the sphere in the rough fitting results deviates from the real center of the sphere in space. Let the rough estimated center of the sphere be $S_{best}$ and the actual center of the sphere be $S_{correct}$. Two coordinate systems that can be translated and overlapped are established, with the two centers of the sphere acting as the origins. The corresponding spatial distribution is presented in Figure 2. The black system is the XYZ coordinate system of the actual center of the sphere $S_{best}$ while the blue system is the XYZ coordinate system of $S_{correct}$. For the coordinate system containing $S_{best}$, a plane composed of any two coordinate axes is used as the interface, and projections are performed in two directions to obtain two two-dimensional circular point clouds.

**Figure 3:**
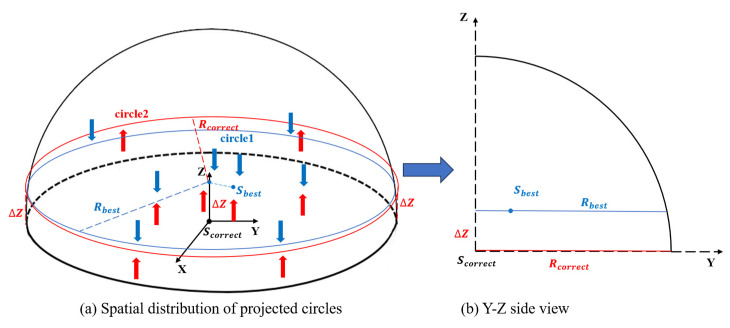
Process of obtaining the correction value in the Z-axis direction (part (**a**) is a three-dimensional space display and part (**b**) is a two-dimensional side-view display).

The spatial positions of the two two-dimensional projected circles are shown in Figure 3a. The red circle, circle2, is the projection formed by the point whose Z-axis value is less than the actual Z-axis value. The red arrow represents the upward projection process. The same is true for the blue circle, circle1. The upper part of the projection can be recorded as circle1 with a radius of $R_{best}$, while the lower part can be recorded as circle2 with a radius of $R_{correct}$. Figure 3b presents a side view of Figure 3a, which more clearly shows the geometric relationships. The correction value ∆Z in the Z-axis direction can be obtained based on the geometric relationship in Equation (8).

(8) $$\Delta Z=\sqrt{abs(R_{best}^{2}-R_{correct}^{2})}$$

To obtain the circumscribed contour points of the two projected two-dimensional circles, we first select the point furthest from the rough estimate as the outer contour start point, which is denoted as $A_{1}$. The furthest point from the center of the rough estimate sphere $S_{best}$ must be on the outer contour. The vector pointing to the point $A_{1}$ from the center of the sphere is denoted as $I_{S1}$. In these circular point cloud data, we consider the initial direction to each point to be a vector $I_{12}$ and determine the point $A_{2}$ that minimizes the angle between the vectors $I_{S1}$ and $I_{12}$. We denote this angle as $\theta_{12}$, which can be derived from the angle between the two vectors, as shown in Equation (9).

(9) $$\theta_{12}=acos(I_{S1}\cdot I_{12}/\left\|I_{S1}\right\|\left\|I_{12}\right\|)$$

**Figure 4:**
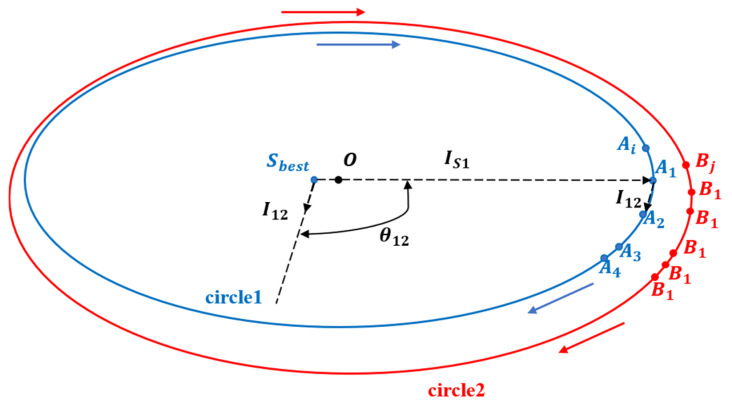
Process of finding the contour points of a two-dimensional circle (the direction of the arrow indicates the direction in which the contour points are found).

The outer contour points are then searched in addition to $A_{2}$ to find $A_{1}$, $A_{2}$, $A_{2}$…, $A_{i}$. Using these points to fit a circle, we can derive the fitting radius values $R_{best}$ and $R_{correct}$ of the two-dimensional point cloud. This process is illustrated in Figure 4.

The correction values ∆X and ∆Y in the X-axis and Y-axis directions can be obtained similarly. Then, the correction value ∆correct for all rough fitting sphere center coordinates can be obtained as shown in Equation (10).

(10) $$\Delta correct=\left(\Delta X,\;\Delta Y,\;\Delta Z\right)$$

The coordinates of the center of the sphere after the rough fitting of RANSAC are added to the correction value ∆correct to obtain the corrected coordinates of the center of the sphere after fitting and are recorded as $S_{O}$. The radius value is calculated using the LS algorithm based on the obtained correction value. The distance from each point in the spherical point cloud to the corrected center of the sphere $S_{O}$ is then calculated, and the distance is subtracted from the radius to obtain the residual. If the absolute value of the residual is too large for a given point, then the point is considered an interference point and removed. The main purpose of this step is to correct the coordinates and radius of the center of the sphere after the rough fitting to minimize the deviation between the center of the sphere fitted by RANSAC in the first step and the actual center of the sphere. The implementation of this process is presented in Algorithm 1.
**Algorithm 1** Projection correction algorithm**Input:** *T*: percentage of the number of rejected point clouds; $P_{in}$: input point cloud;**Output:** fitted parameters after spherical center correction; $R_{correct}$: Radius of the corrected model; $Sphere_{correct}$: coordinates of the corrected model; $p_{out}$: Point cloud after removal of interference points;1: set $T,\;P_{in}\;$ put in;2: **for**  i=0; i <3; i ++  **do**3:  the point cloud data is projected on the plane to get two circles,  circle1,circle 2;
4:  calculate the angle to get the outer contour, denoted by the set $A_{i};$
5:  calculate the radii of the upper and lower circular projections, denoted $\;R_{1}\;,R_{2};$
6:  calculate the correction in the direction perpendicular to the plane;7: **end for**8: calculate $R_{correct};$
9: output the best model, $R_{correct},\;Sphere_{correct},\;P_{correct}\;;$


### 2.3. Accurate Fitting of Parameters

The coordinates of the sphere center after projection filtering are closer to the actual value than the rough fitting parameters of RANSAC. To obtain fitting parameters accurately, we first set the minimum voxel value $d_{Voxel}$. Because there are too many options for selecting samples from the point cloud, recursive RANSAC is applied to sample the point cloud model after removing interference points to obtain the fitting sphere center point set. Recursive RANSAC introduces the repeated use of random sampling, as well as the continuous updating and resampling of the internal point set, making the model fitting more robust.

After sampling, owing to the location of the points and the existence of interference points, multiple clusters deviating from the actual center of the sphere were formed, so the K-means clustering algorithm was applied to find the largest cluster. The K-means clustering algorithm minimizes the objective function *J* in Equation (11). The specific calculation process is defined in Equations (12) and (13).

(11) $$J=\sum_{n=1}^{N}\sum_{k=1}^{K}r_{nk}{\left\|X_{n}-\mu_{k}\right\|}^{2}$$

(12) $$r_{nk}=\left\{\begin{array}{ll} 1 & \mathrm{if}\;k=\arg\min_{j}{\left\|X_{n}-\mu_{j}\right\|}^{2}\\ 0 & \mathrm{otherwise} \end{array}\right.$$

(13) $$u_{k}=\frac{\sum_{n}r_{nk}X_{n}}{\sum_{n}r_{nk}}$$

**Figure 5:**
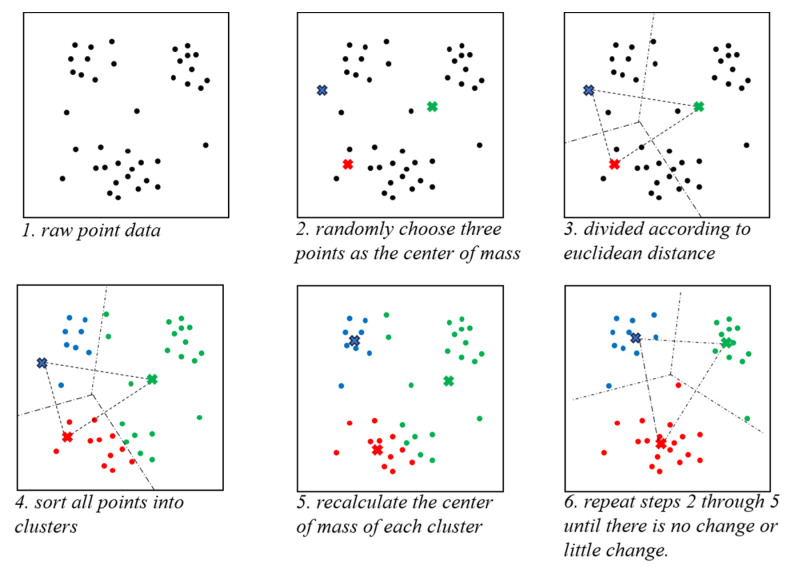
K-means clustering process.

The concept of K-means clustering is to select K samples from a set of clustered samples, and then traverse all samples and calculate the distance between each sample and the K samples. The sample is classified into the category with the smallest distance. In this manner, all samples are assigned to their respective categories. The centroids of the samples in the K categories are then recalculated and the algorithm returns to the first step to continue iteration, repeating the above steps until the centroids of the samples in the K categories no longer move or move very little. Figure 5 illustrates the implementation process of this clustering algorithm.

**Figure 6:**
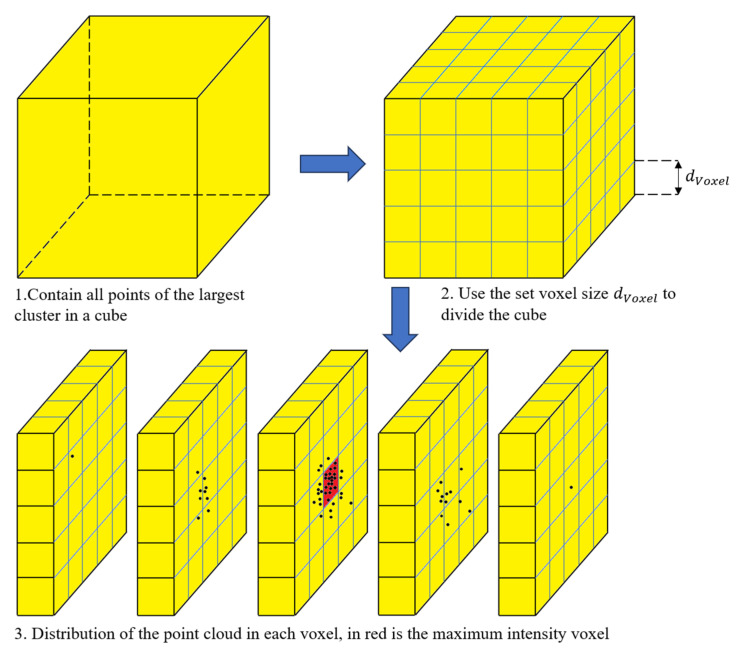
Process of finding the maximum-intensity voxel.

The distribution of the point cloud in the largest cluster is still dense in the center. Therefore, we process the largest cluster. Specifically, we predefine the parameter $d_{Voxel}$, divide the coordinate value of each point by the minimum voxel value and round it, multiply it by the parameter value, and then add one to the value of the voxel to which the point belongs. A conceptual visualization of this process is presented in Figure 6. The closer the voxel value is to the actual center of the sphere, the larger the voxel value and the more voxels have non-zero values. Therefore, we select the voxel with the largest value as the maximum-intensity voxel, set the distance weight *W* in Equation (16), and weight the voxel according to the distance weighting function. The parameter *d* represents the Euclidean distance from each point to the center of the maximum-intensity voxel. In this manner, both the weight of the dense area and the influence of the coordinates of the surrounding sparse points are considered.

(14) $$a=\frac{5\sqrt{3}}{2}-\sqrt{\frac{\sigma^{2}}{76}-0.25}$$

(15) $$b=0.5-5\sqrt{3}\cdot \sqrt{\frac{\sigma^{2}}{76}-0.25}$$

(16) $$W=\left\{\begin{array}{ll} b+\sqrt{R^{2}-{\left(\sigma -a\right)}^{2}} & if\;d<5\sqrt{3}\cdot d_{Voxel}(\sigma \geq 38)\\ 0 & else \end{array}\right.$$

**Figure 7:**
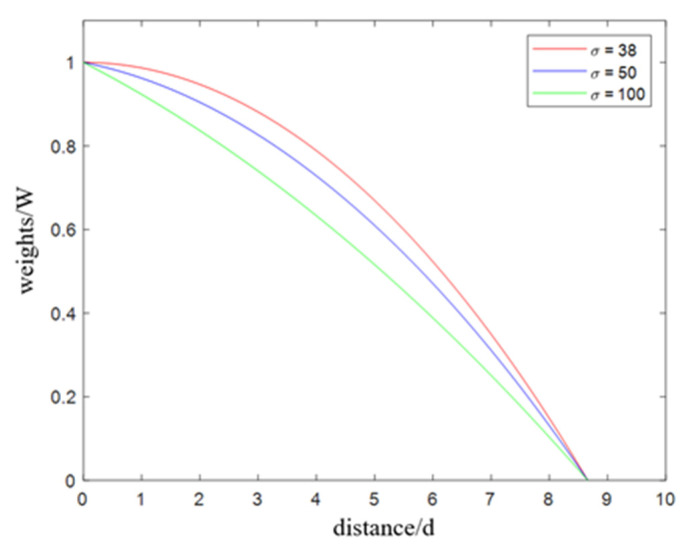
Effect of the parameter *σ* on the decay of weights (the ordinate is the weight value, and the abscissa is the distance from each point to the maximum-intensity voxel).

**Figure 8:**
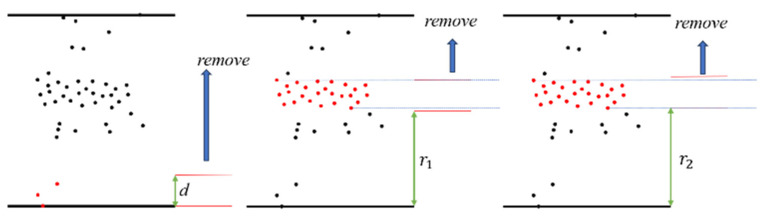
Procedure for finding the radius r (the direction of movement is bottom-up (i.e., away from the center of the sphere)).

In the formulas above, the parameter σ is used to control the attenuation rate. Combined with the information in Figure 7, one can see that the larger the value of σ, the faster the weight decays. After the corrected sphere center coordinate value is obtained according to the distance-weighted formula, the sphere radius is obtained as shown in Figure 8. The steps are to set the interval width *d* and start from the point closest to the fitting sphere center coordinate and move outward from the sphere until the number of points in the interval is maximized, and the distance is recorded as $r_{1}$. When the number of point clouds in the red interval in the figure decreases, the distance is recorded as $r_{2}$. The radius r calculation formula is defined in Equation (17). The purpose of this step is to obtain accurate fitting parameters. The corresponding pseudo-code is presented in Algorithm 2.

(17) $$r=\frac{\left(r_{1}+r_{2}\right)}{2}+d$$

**Algorithm 2** Accurate fitting of parameters**Input:** $P_{in}$: input point cloud; σ: attenuation factor; k: number of K-Means clustering clusters; $d_{Voexl}$: minimum voxel resolution set;**Output:** accurately fitting the parameters of the ball; $R_{correct}$: precisely fitted radius;$Sphere_{correct}$: precisely fitted coordinates;1: set $\;P_{in}\;$ put in;2: RRANSAC sampling grouping to obtain the set of fitted spherical center points;3: K-Means clustering to find maximal clusters;4: somatization of the largest clusters based on $d_{Voexl}$;5. correction with distance weighting function;6: output the best model, $R_{correct},\;Sphere_{correct};$


## 3. Results

This section verifies the feasibility of our method by combining random spherical point clouds generated through simulation experiments with two CT datasets to derive the two important parameters of sphere fitting, namely the sphere center coordinates and sphere radius. For the sphere center coordinate fitting, we initially conducted simulation experiments and verified the results using CT cylinder-ball reference object experiments. The sphere radius fitting was verified through simulation experiments and CT ball-plate standard experiments. During our experiments, the fitting data from algorithms such as RANSAC and PC-RANSAC were measured at the same time to facilitate data comparison.

### 3.1. Simulation Data Experiment

**Figure 9:**
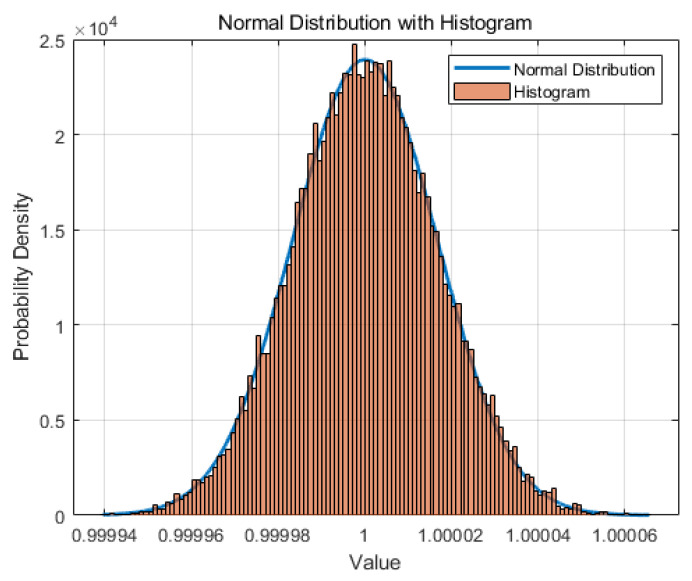
Normal distribution of deviation (the blue curve is the normal distribution curve, and the orange bars form a distribution histogram of the deviation).

We first generated a spherical point cloud through simulation and then used the point cloud data to perform geometric parameter fitting experiments. The spherical point cloud consisted of 25,000 points, of which 2000 were interference points. The deviation between the Euclidean distance from other points and the center of the sphere, and the set radius was within 0.005%. The preset center coordinates and the positions of all points were randomly generated, and the deviation from the points to the preset sphere surface followed a normal distribution, as shown in Figure 9. The blue curve in the figure is a normal distribution curve, and the orange bars form a distribution histogram of the deviation.

**Figure 10:**
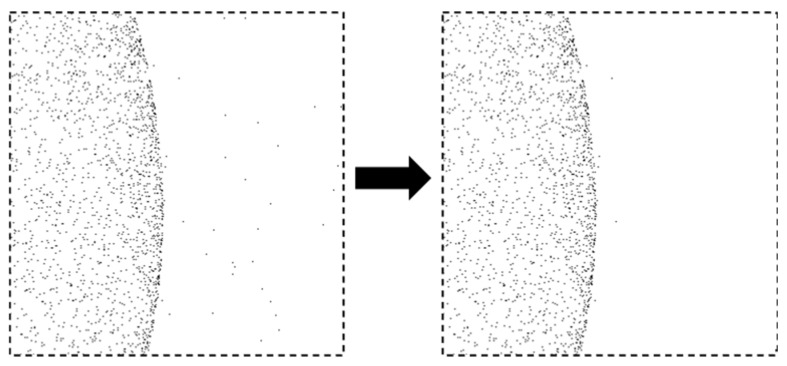
Comparison of the point clouds before and after statistical outlier removal filtering.

Figure 10 presents the changes in the point cloud model before and after RANSAC fitting using statistical outlier removal filtering, where one can see that the number of outliers was reduced.

**Figure 11:**
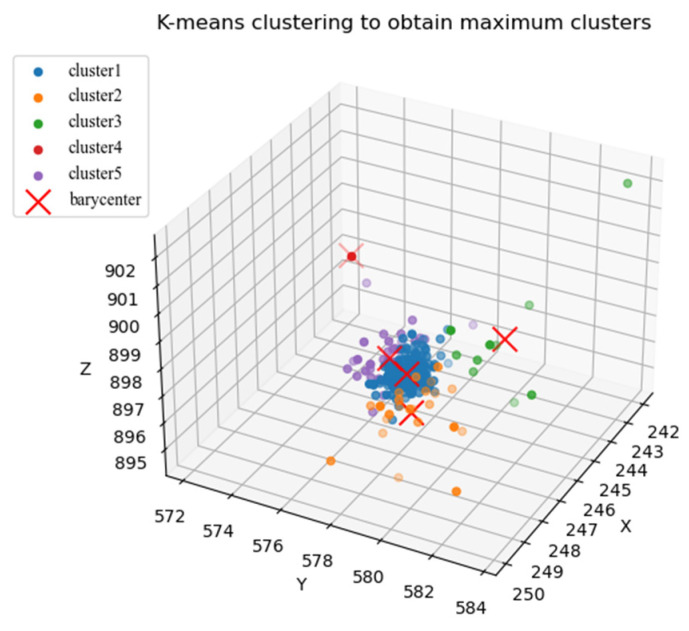
K-means clustering based on fitted spherical center point sets (the cross symbol indicates the barycenter).

Through the projection filtering operation in the second step, the sphere center correction distance was obtained. Then, the distance residual from each point to the corrected sphere center was calculated, and 0.27% of the points were eliminated according to the residual size and three-sigma rule. The value of 0.27% was obtained according to the normal distribution. After sampling fitting, the fitted sphere center point set was obtained. Through K-means clustering, the fitted sphere center points were improved and divided into five categories. Figure 11 presents the clustering results, where the blue cluster is the largest cluster identified through clustering.

In the simulation experiments, the true value was the set value of the simulated data, and the average value of multiple measurements was calculated as the final value. Comparisons of the fitted value and true value after each step of PK-RANSAC are presented in Table 1. Step 1, Step 2, and Step 3 in the table correspond to the three steps of PK-RANSAC. Each step yields improvements compared with the previous step and PK-RANSAC improves upon RANSAC, yielding values closer to the true value. Table 2 presents the reduction in deviation in each step.

To evaluate the repeatability and feasibility of the PK-RANSAC fitting algorithm, 20 spherical point clouds were fitted by changing the coordinates of the spherical center, radius, and random normal distribution of spherical surface points. The deviation between the Euclidean distances of all points and the center of the sphere, and the set radius was within 0.005%. The value of the sphere radius was randomly set to 10 or 20 and the fitting experimental results are presented in Table 3. Because the deviation was within 0.005%, for the spherical point cloud with a preset radius of 10, the deviations of the two geometric parameters (fitting radius and coordinate values) should be less than 0.05. Similarly, for a radius of 20, the deviations should be less than 0.1. As shown in Table 3, the fitting parameters obtained by the RANSAC algorithm deviate too far from the true value, whereas the fitting of the PK-RANSAC algorithm is significantly improved.

### 3.2. Actual Measurement of CT Data Experiment

**Figure 12:**
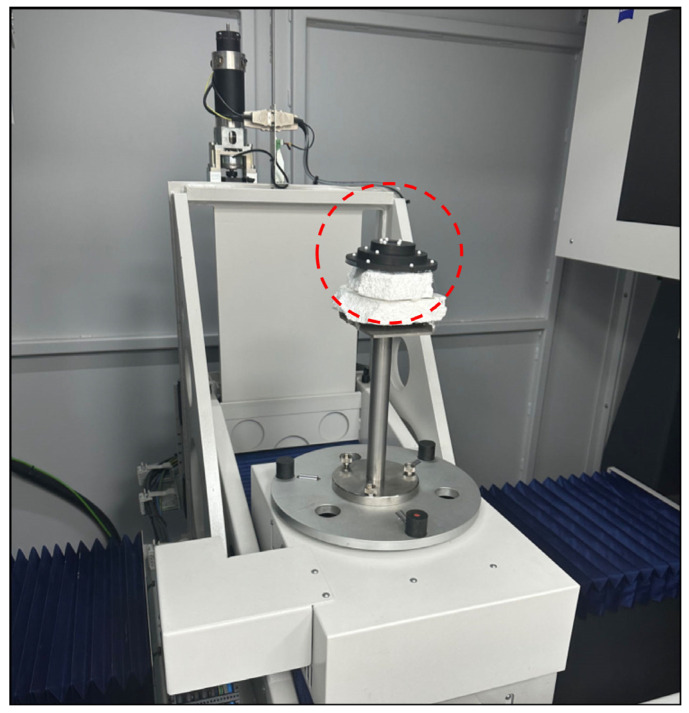
Stepped ball-plate standardizer placement (the step-type ball-plate standard is inside the red circle).

The true values of the geometric parameters for the real CT experiment were measured using a high-precision CMM F25 instrument from the China Academy of Metrology and Science, which has a measurement accuracy of 0.25 μm. The actual raw projection data were obtained using a high-precision X-ray tomography machine. The principle of CT measurement is based on the different attenuation coefficients of materials for X-rays, and images are reconstructed using multiple projection images of X-rays passing through the detector from different angles of the object under test. The first measured CT dataset was collected from a cylinder-ball reference object, where the ball was made of a different material than the cylinder. The main purpose of this experiment was to evaluate the performance advantages and disadvantages of the PK-RANSAC fitting algorithm for determining the radius. The second CT dataset consisted of a stepped ball-plate standard, comprising a black carbon brazed dimensional sphere plate with 15 white ceramic spheres. This experiment aimed to evaluate the merits of PK-RANSAC in terms of fitting spherical coordinates by optimizing the fitting of the spherical centroid distance parameter. The stepped ball-plate standard was fixed to the instrument as shown in Figure 12. The radii and coordinates of the spheres, as well as the sphero-centric distances between spheres, were obtained by fitting the geometrical parameters of the 15 spheres.

**Figure 13:**
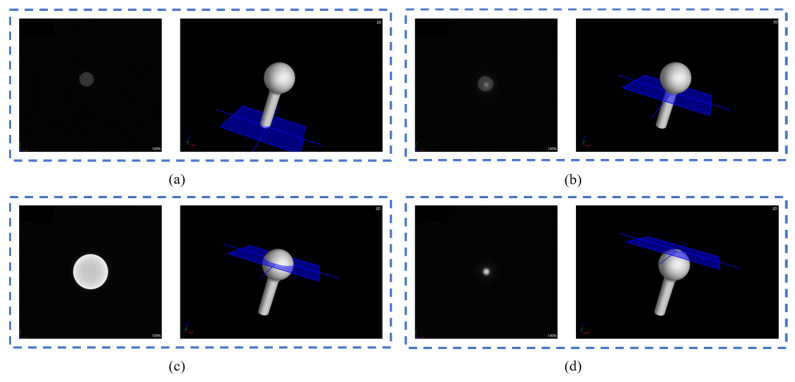
Slices of cylinder-ball reference object CT reconstructed volume data (slice order: **a**–**d**).

**Figure 14:**
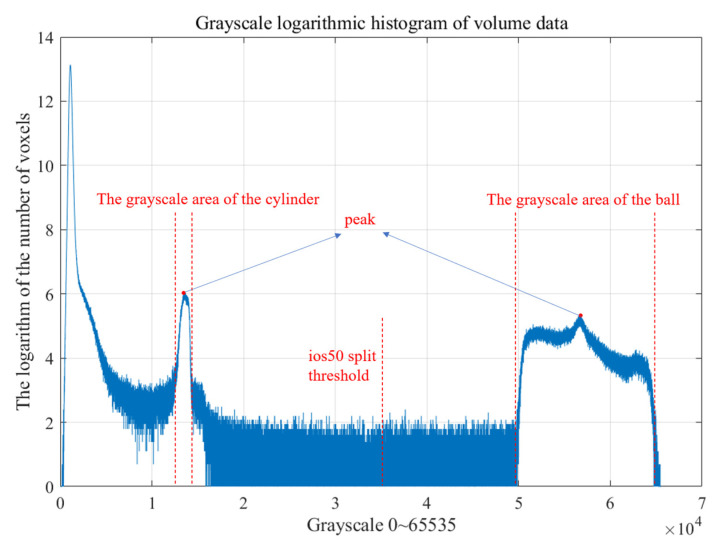
Voxel logarithmic histogram of cylinder-ball reference object grayscale values.

For the cylinder-ball reference object experiment, the projection data were reconstructed, and the reconstructed volume data slices are presented in Figure 13. All voxel values of the reconstructed volume data are in the grayscale range of 0~65,535. Figure 14 presents a logarithmic intensity histogram of the volume data voxels. The horizontal axis represents the grayscale value, and the vertical axis is the logarithm of the number of voxels corresponding to the grayscale value. The first peak area represents the grayscale value of the cylinder, while the second represents the grayscale value of the sphere. Based on ios50, threshold segmentation was performed to derive the median of the grayscale values corresponding to the two peaks and use this value as the segmentation threshold. The mesh model and point cloud were obtained using the gridding algorithm. Because the adhesion of the lower half of the sphere to the cylinder affects the fitting process of the sphere, the cropbox filter was used to remove the adhesion area. The experimental results are presented in Table 4, according to which the PK-RANSAC fitting value is closer to the true value compared to the RANSAC fitting value.

**Figure 15:**
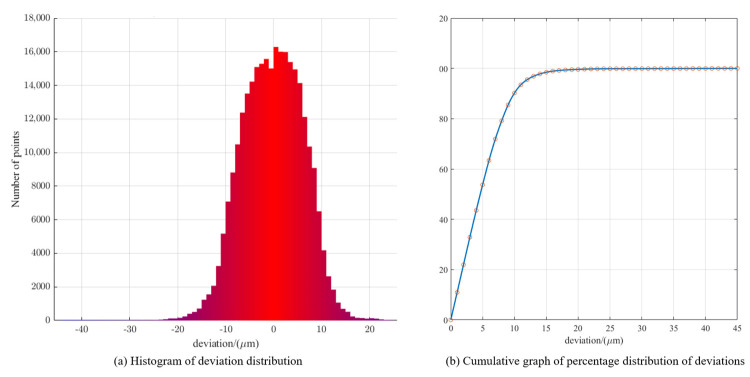
(**a**) Fitting deviation histogram and (**b**) percentage accumulation graph.

To verify the performance of PK-RANSAC further, the deviation from each point to the surface of the fitting sphere was calculated after fitting. The formula for the sphere center deviation is defined in Equation (18), and a distribution histogram of the deviation and percentage accumulation diagram of the deviation are presented in Figure 15. Figure 15a reveals that the distribution of the deviation is somewhat similar to the normal distribution in shape, and the smaller the absolute value of the deviation, the more points are distributed. In Figure 15b, one can see that points with a deviation within 10 μm account for 90.204% of all points.

(18) $$\Delta D=abs(R_{fit}-R_{real})$$

Mean squared error (MSE) refers to the average value of the sum of squared errors. This evaluation index is sensitive to outliers, and considering square values further increases the impact of errors. The MSE calculation formula for a single cylinder-ball reference object point cloud is defined in Equation (19), and an MSE value comparison between the two methods is presented in Table 5. One can see that the MSE value of PK-RANSAC is significantly smaller than that of RANSAC.

(19) $$\left\{\begin{array}{l} d_{i}=sqrt({\left(x_{i}-x_{real}\right)}^{2}+{\left(y_{i}-y_{real}\right)}^{2}+{\left(z_{i}-z_{real}\right)}^{2})-r_{real}\\ MSE=\frac{1}{n}\sum_{i=1}^{n}{\left(d_{i}\right)}^{2} \end{array}\right.$$

**Figure 16:**
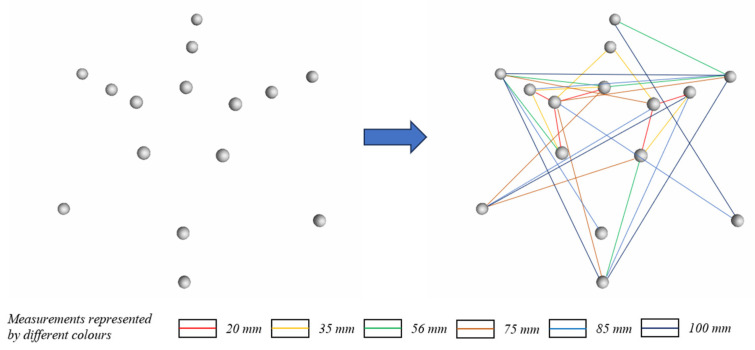
Fifteen spheres and thirty sphere center distances (different colors represent different values).

The test object in the second CT experiment was a three-layer stepped ball-plate standard with a total of 15 spheres, and we measured 30 sphere center distances. Figure 16 presents the spatial distribution of the 15 spheres on the stepped standard. The lines in the figure represent the 30 measured sphere center distances. The measured parameters of the 30 sphere distances were divided into six values according to their magnitude. Each value had five directions, and different colors represented different values. The experimental results are presented in Table 6, where sphere pairs are represented by numerical labels connected by underscores. We used RANSAC, PC-RANSAC, and PK-RANSAC to fit the 30 sphere center distances and compare them with the true values. PK-RANSAC significantly outperformed RANSAC. The average deviation of RANSAC was 25.41 μm, that of PC-RANSAC was 11.20 μm, and that of PK-RANSAC was 1.91 μm.

## 4. Discussion

In this section, we consider five different algorithms to present a comparative analysis based on the three experiments described in the previous section. These algorithms include the LS method, RANSAC, improved RANSAC (PC-RANSAC), TLS, and the proposed PK-RANSAC. The LS algorithm minimizes total error by constructing a linear equation system to obtain fitting parameters. Although this approach is simple and easy to implement, it is easily affected by outliers and noise, resulting in poor fitting results. RANSAC is an efficient iterative method that can effectively fit model parameters under conditions with more noise and outliers. PC-RANSAC combines the advantages of LS and RANSAC to improve fitting accuracy further. TLS introduces a truncation function based on LS and enhances robustness by effectively handling outliers.

**Figure 17:**
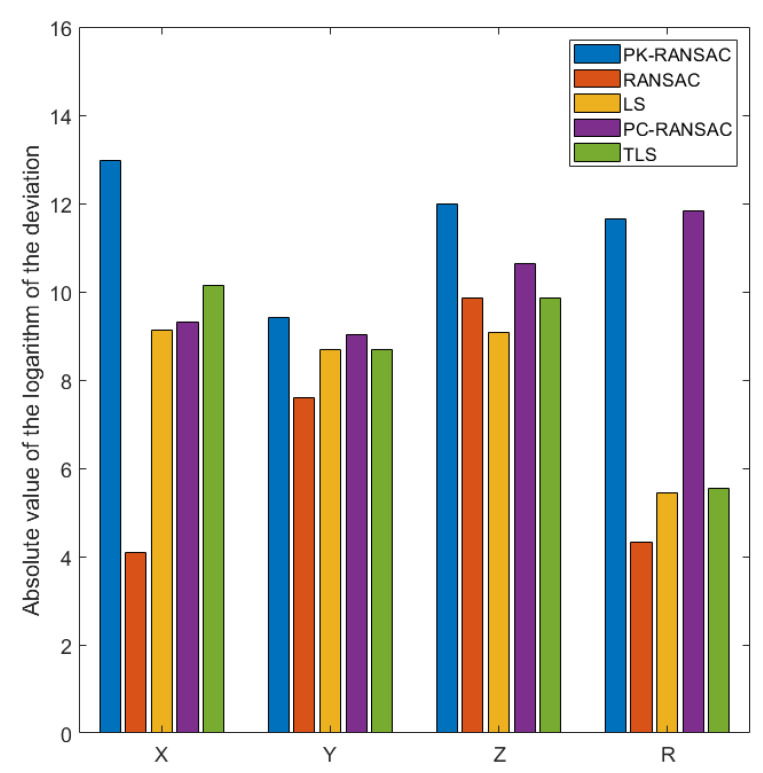
Comparison of multiple methods on simulation experiment data. The vertical axis represents the absolute value of the logarithm of the deviation, meaning larger values represent smaller deviations of the fitted geometrical parameters; the horizontal axis represents the three parameters of the spherical center coordinates (X, Y, Z) and the radius of the sphere.

The fitting results for the simulation data are presented in Figure 17. To show the performance differences between different algorithms clearly, the deviation values are presented in the form of logarithmic absolute values as the ordinate. This visualization amplifies differences in deviation, highlighting slight differences in algorithm performance. The larger the value of the ordinate, the closer it is to the set true value. PK-RANSAC provides significant advantages for fitting the two key parameters of the sphere center coordinates and sphere radius. Specifically, for both parameters, the deviation values of PK-RANSAC are significantly smaller than those of the other four methods, demonstrating that it has greater stability and accuracy when handling complex point cloud data. In contrast, although TLS and PC-RANSAC exhibit obvious improvements in terms of handling outliers and noise compared with LS and RANSAC, PK-RANSAC still provides superior fitting accuracy. This demonstrates that PK-RANSAC improves overall performance by combining the advantages of RANSAC and projection filtering, providing outstanding performance in complex environments. Overall, these results indicate that the application of PK-RANSAC to industrial CT measurement has great potential and can provide a more reliable solution for spherical point cloud fitting.

**Figure 18:**
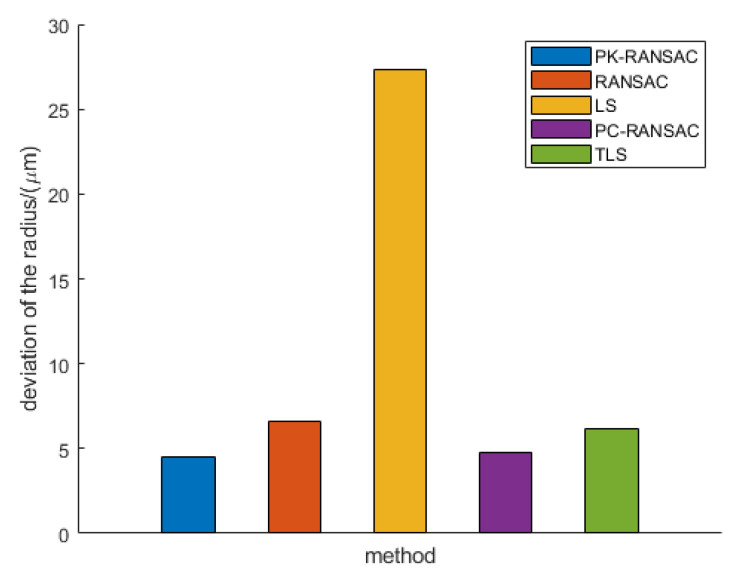
Comparison of multiple methods for cylinder-ball reference object fitting experiments.

In the CT cylinder-ball reference object experiment, we considered the same five methods to conduct a comparative analysis of the parameter of sphere radius. Figure 18 presents the deviations of the five point cloud algorithms when fitting the radius compared with the true value. The ordinate represents the deviation between the fitting radius and the true value. The smaller the value, the closer the fitting result is to the true value, indicating better performance. There are inevitable errors in the reconstruction process of CT, and certain deviations are introduced in the conversion from reconstructed volume data to a mesh model. However, the mesh model used by all fitting algorithms is the same, so each method has the same inherent deviation between the fitted radius and true value. This deviation can be considered a constant, so the closer the fitting result is to the true value, the better the performance of the algorithm. PK-RANSAC provides the best radius fitting performance with the smallest deviation, demonstrating excellent fitting accuracy and robustness. In contrast, the LS method is easily affected by outliers, and the fitting results contain large deviations, demonstrating the disadvantage of insufficient robustness. Additionally, although RANSAC, PC-RANSAC, and TLS improved fitting accuracy to a certain extent, they still failed to surpass the superior performance of PK-RANSAC. These results demonstrate that PK-RANSAC can effectively reduce the deviation introduced by noise and outliers when processing complex CT cylinder-ball reference object data, yielding a fitting radius close to the true value. This performance improvement is of great significance for the precision of CT measurement, especially in industrial applications that require high-precision fitting. Based on the results of our comparative analysis, it can be concluded that PK-RANSAC provides significant advantages for sphere radius fitting and is superior to the other four methods in terms of fitting accuracy and robustness, demonstrating that PK-RANSAC is not only advanced in theory but also exhibits good applicability and reliability in practical applications.

**Figure 19:**
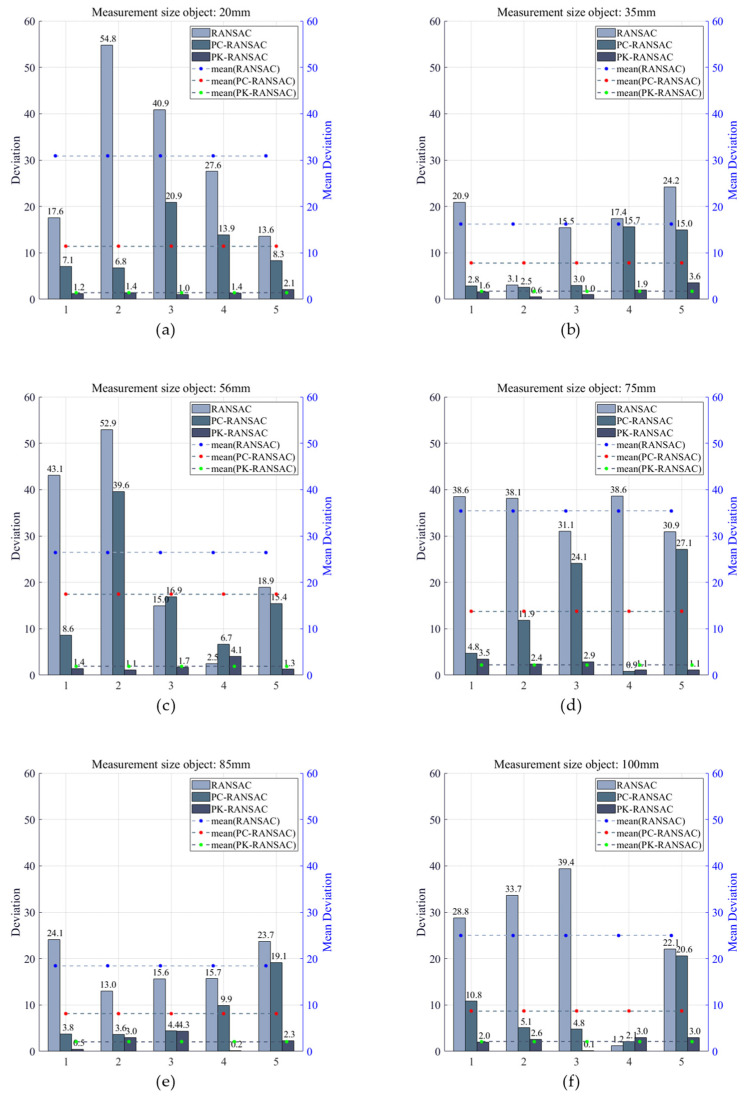
Measurement object size. (**a**–**f**) represent fitting experiments for six values and thirty spherical center distances. The vertical and horizontal axes represent deviations and spherical center distances, respectively, and the unit of deviation is μm.

For the CT ball-plate standard experiment, we selected three algorithms, namely, RANSAC, PC-RANSAC, and PK-RANSAC, for comparative analysis to evaluate their performance in terms of fitting spherical center coordinates. Figure 19 presents the deviations of 30 spherical center distances in the second group of CT-measured data. The numerical data are summarized in Table 6. The horizontal axis represents the spherical center distances, while the vertical axis represents the deviation values. The three lines of different colors in the figure represent the fitting means of the three methods. Owing to its randomness and instability, RANSAC leads to large deviations between the fitting results and true values, indicating that it does not perform well when processing CT data. This instability of RANSAC may lead to a significant decrease in fitting accuracy on some complex point cloud data, negatively affecting the accuracy of geometric parameters. Although PC-RANSAC improves the robustness of fitting to a certain extent, it is still inferior to PK-RANSAC in terms of deviation control. PK-RANSAC exhibits excellent stability and accuracy for fitting the spherical center distances, and its fitting deviations are all within 5 μm, with an average deviation of only 1.91 μm. This demonstrates that PK-RANSAC not only has superior robustness for the processing of noise and outliers, but also has significant advantages in terms of the fitting accuracy of geometric parameters. These results further demonstrate the application potential of PK-RANSAC in industrial CT measurement and the provision of reliable technical support for high-precision measurement needs.

From our discussion of the three experiments above, several important conclusions can be drawn. First, although the LS method provides a simple and direct fitting method in theory, its robustness is poor and it is easily disturbed by outliers, which significantly reduces the reliability of the fitting results. Because the LS method does not effectively handle noise and outliers, it exhibits obvious deficiencies in complex datasets. Second, although RANSAC improves noise resistance to a certain extent through iterative processing, its inherent randomness produces results with greater uncertainty, especially for large-scale point cloud data, rendering it difficult to ensure the repeatability of results. This lack of stability makes RANSAC inappropriate for certain practical applications, especially in scenarios with high precision requirements. In contrast, PK-RANSAC demonstrates excellent performance. Its innovation lies in the strategic integration and enhancement of two methods, which improves robustness and repeatability. The workflow of PK-RANSAC is divided into three steps, and this multi-stage processing approach not only enhances the robustness of the algorithm but also effectively reduces the uncertainty of calculations. Consequently, PK-RANSAC achieves a high degree of fitting accuracy and stability in the presence of noise and outliers, allowing it to provide more reliable and accurate results on complex industrial CT measurement tasks.

In summary, PK-RANSAC not only overcomes the limitations of the LS method and RANSAC in terms of processing complex point cloud data, but also ensures the high accuracy and stability of fitting results through a multi-step optimization process. This makes PK-RANSAC a superior algorithm with broad application prospects in point cloud fitting, which can provide strong technical support for high-precision measurement.

## 5. Conclusions

In order to better address the issue of spherical point cloud target fitting (STF), we propose an improved PK-RANSAC based on the RANSAC. This new approach significantly outperforms RANSAC in terms of fitting accuracy and also surpasses the robustness of least squares methods and their variants. Through validation with simulated data, PK-RANSAC demonstrates exceptional feasibility and advantages. In experimental tests, the cylinder-ball reference object data experiments confirm the high precision of PK-RANSAC in fitting spherical radii, while experiments with stepped ball-plate standard data showcase its outstanding performance in fitting sphere center coordinates. The results indicate that the integration of projection filtering and K-means clustering methods achieves remarkable accuracy and robustness, offering significant advantages over traditional algorithms. However, the algorithm currently requires a longer processing time, and improving its efficiency will be a focus for future efforts. PK-RANSAC is not only applicable for enhancing fitting accuracy in the field of CT imaging, but also for point cloud registration. This algorithm holds broad application prospects in industrial inspection, reverse engineering, and materials science.

## Figures and Tables

**Table 1:** Comparison of fitted values after each step with true values.

Parametric	Step 1	Step 2	Step 3	True Value
X	246.917175	246.936550	246.933054	246.933484
Y	578.279846	578.304406	578.303259	578.303252
Z	897.144836	897.113507	897.077428	897.076754
R	10.049875	9.998437	9.999055	10.000000

**Table 2:** Deviation at each step.

Parametric	Step 1–Step 2	Step 2–Step 3	Step 1–Step 3
X	0.013243	0.002636	0.015879
Y	0.022252	0.001147	0.023399
Z	0.031329	0.036079	0.067408
R	0.048312	0.000618	0.048930

**Table 3:** Multiple spherical point cloud experiments (Group: Number of groups in the experiment; P: Geometric information parameters of the sphere).

Group	P	RANSAC	PK-RANSAC	Real Value	Group	P	RANSAC	PK-RANSAC	Real Value
1	X	246.917175	246.933054	246.933484	11	X	743.550598	743.568272	743.561821
	Y	578.279846	578.303259	578.303252		Y	306.055450	306.016250	306.014524
	Z	897.144836	897.077428	897.076754		Z	882.056213	882.043980	882.044992
	R	10.049875	9.999055	10.000000		R	20.046820	20.000345	20.000000
2	X	561.129333	561.129555	561.130878	12	X	704.275024	704.301214	704.301318
	Y	195.709412	195.725723	195.725104		Y	867.981934	868.012446	868.012579
	Z	226.497665	226.502046	226.501991		Z	26.350420	26.769622	26.769830
	R	20.078751	19.998826	20.000000		R	9.643651	10.000120	10.000000
3	X	381.793457	381.769778	381.774528	13	X	572.365173	572.703407	572.702488
	Y	285.973022	285.936395	285.933115		Y	834.539856	834.617653	834.619924
	Z	98.477936	98.480072	98.481401		Z	728.508972	728.564828	728.564621
	R	19.986713	19.999574	20.000000		R	10.024969	9.999402	10.000000
4	X	786.689819	786.673251	786.675099	14	X	889.814880	889.854685	889.858171
	Y	254.344620	254.254536	254.262859		Y	671.338440	671.589943	671.590496
	Z	709.607544	709.591002	709.584786		Z	141.074600	141.017180	141.016547
	R	20.062403	19.999986	20.000000		R	19.990623	19.999695	20.000000
5	X	493.628815	493.629719	493.630735	15	X	883.981506	884.086524	884.087031
	Y	922.256470	922.255856	922.255376		Y	415.568085	415.715176	415.711885
	Z	59.103256	59.186219	59.184562		Z	505.630524	505.647207	505.650742
	R	10.062305	9.999053	10.000000		R	20.112185	20.000311	20.000000
6	X	947.520935	947.542646	947.540757	16	X	105.581985	105.183584	105.183946
	Y	481.574310	481.964760	481.967207		Y	766.359253	766.367362	766.366602
	Z	315.237671	315.274308	315.274122		Z	708.879089	708.905112	708.907239
	R	10.000000	10.000708	10.000000		R	10.198039	10.000627	10.000000
7	X	639.219604	639.264039	639.264681	17	X	248.298431	248.303789	248.304023
	Y	28.855110	28.930647	28.931308		Y	132.508316	132.527841	132.525323
	Z	858.563965	858.567802	858.567937		Z	100.069572	100.038094	100.036081
	R	9.905807	9.999752	10.000000		R	10.016003	9.999272	10.000000
8	X	147.419312	147.413612	147.415414	18	X	593.301453	593.198806	593.199986
	Y	836.551270	836.549566	836.547329		Y	581.203674	581.386051	581.384344
	Z	134.673981	134.687489	134.689662		Z	806.116821	806.239197	806.240295
	R	20.020302	19.999798	20.000000		R	9.924717	9.999868	10.000000
9	X	542.255432	542.241369	542.243279	19	X	522.112244	521.945001	521.945154
	Y	333.036255	333.098283	333.102226		Y	323.894165	323.514885	323.515338
	Z	548.272949	548.264637	548.264175		Z	976.824036	976.965890	976.967813
	R	20.034346	19.999546	20.000000		R	10.142732	9.999370	10.000000
10	X	810.159546	810.166847	810.172006	20	X	656.082031	655.977859	655.979157
	Y	737.505981	737.509693	737.515096		Y	641.028137	641.032843	641.035319
	Z	136.618729	136.559892	136.559440		Z	789.694458	789.657126	789.660352
	R	19.927996	19.999055	20.000000		R	20.018742	19.999298	20.000000

**Table 4:** Comparisons of RANSAC and PK-RANSAC radius fitting values with the true value.

Geometric Parameter	RANSAC (μm)	PK-RANSAC (μm)	Real Value (μm)
Radius value	5128.45	5130.55	5135.01

**Table 5:** Comparison of MSE values of fitting methods.

Unit: μm^2^	RANSAC	PK-RANSAC
MSE value	48.1012	41.2714

**Table 6:** Comparison of polar coordinate fitting values for CT data (μm).

Measurements	Serial Number	Name	RANSAC	PC-RANSAC	PK-RANSAC	Real Value
20	1	5_8	20,083.72	20,108.38	20,100.08	20,101.28
	2	6_8	20,059.21	19,997.61	20,005.80	20,004.41
	3	7_9	20,047.70	20,109.51	20,089.57	20,088.58
	4	8_10	20,014.73	20,056.16	20,040.95	20,042.30
	5	9_11	20,032.60	20,054.53	20,044.05	20,046.19
35	1	4_8	35,005.33	34,981.61	34,982.84	34,984.45
	2	4_9	35,011.44	35,011.96	35,013.93	35,014.50
	3	5_6	35,122.75	35,104.30	35,106.30	35,107.28
	4	5_10	35,111.73	35,110.02	35,092.39	35,094.34
	5	7_11	35,061.41	35,070.59	35,082.02	35,085.59
56	1	1_6	56,369.33	56,317.61	56,324.81	56,326.23
	2	1_10	56,327.69	56,314.37	56,273.66	56,274.76
	3	2_3	56,048.36	56,046.38	56,061.60	56,063.32
	4	3_6	56,376.58	56,372.45	56,375.03	56,379.11
	5	11_15	56,114.47	56,110.97	56,094.29	56,095.56
75	1	1_9	75,018.04	74,974.72	74,975.99	74,979.47
	2	3_8	75,024.88	74,974.91	74,984.36	74,986.79
	3	6_12	75,120.82	75,113.87	75,092.66	75,089.77
	4	8_15	74,793.93	74,831.70	74,831.42	74,832.57
	5	11_12	75,176.38	75,172.59	75,144.37	75,145.49
85	1	1_13	84,181.63	84,153.74	84,157.04	84,157.51
	2	3_5	84,158.45	84,149.05	84,142.46	84,145.42
	3	7_15	84,054.58	84,034.58	84,034.65	84,038.97
	4	8_14	86,520.92	86,546.48	86,536.80	86,536.61
	5	9_12	86,455.69	86,451.12	86,429.70	86,432.01
100	1	1_3	99,968.40	99,950.47	99,937.68	99,939.65
	2	1_15	99,884.27	99,855.64	99,847.98	99,850.57
	3	2_14	100,147.72	100,191.89	100,186.96	100,187.11
	4	3_15	99,829.75	99,833.07	99,827.96	99,830.94
	5	7_12	99,879.91	99,878.44	99,854.87	99,857.84

## Data Availability

Data will be made available upon reasonable request.
